# Human esophageal fibroblast-derived exosomal miR-21 reduced the cisplatin sensitivity to esophageal carcinoma EC9706 cells

**DOI:** 10.1590/1414-431X2021e11156

**Published:** 2021-08-06

**Authors:** Jiajin Wan, Chunling Niu, Baiyan Wang, Qianqian Han, Yulon Chen, Shuying Feng, Lianhe Yang

**Affiliations:** 1 Medical College, Henan University of Chinese Medicine, Zhengzhou, China; 2 Academy of Chinese Medicine Sciences, Henan University of Chinese Medicine, Zhengzhou, China

**Keywords:** Esophageal carcinoma, Human esophageal fibroblasts, Exosomes, miR-21, Chemosensitivity, Cisplatin

## Abstract

The objective of this study was to investigate the effect of human esophageal fibroblast-derived exosomal miR-21 on cisplatin sensitivity against esophageal squamous EC9706 cells. EC9706 cells were co-cultured indirectly with human esophageal fibroblasts (HEF) or miR-21 mimics transfected-HEF in the transwell system. The exosomes in HEF-culture conditioned medium were extracted by differential ultracentrifugation. EC9706 cells were co-cultured with HEF-derived exosomes directly. The cisplatin sensitivity against EC9706 cells was revealed via half maximal inhibitory concentration (IC_50_) values using MTT assay. The expressions of miR-21, programmed cell death 4 (PDCD4) mRNA, and gene of phosphate and tension homology deleted on chromosome ten (PTEN) mRNA were determined by qRT-PCR. The changes of the protein level were detected using western blot assay. IC_50_ values of cisplatin against EC9706 cells were increased after EC9706 cells were co-cultured with either HEF or exosomes derived from miR-21 mimics-transfected HEF. Following the increased level of miR-21, the mRNA expression and protein levels of PTEN and PDCD4 were decreased in EC9706 cells. The cisplatin sensitivity to EC9706 cells was reduced by HEF-derived exosomal miR-21 through targeting PTEN and PDCD4. This study suggested that non-tumor cells in the tumor micro-environment increased the tumor anti-chemotherapy effects through their exosomes.

## Introduction

Esophageal cancer (EC) is a common malignant tumor in the digestive system, ranking 6th in global incidence of the most common cause of death in cancer patients. Esophageal squamous cell carcinoma (ESCC) accounts for 70% cases of EC globally, and now remains a common pathological type highly prevalent in northern China, called “esophageal cancer belt” (1). Presently, chemotherapy remains the first-line approach for EC treatment and plays an important role in limiting tumor growth before surgery and radiotherapy. In recent decades, although great progress has been made in chemo-therapeutics, its failure due to drug-resistant tumors still occurs frequently in clinical practice. When cisplatin is used to clinically treat EC for a long time, EC cells tend to become resistant to cisplatin treatment (2). Therefore, to study the resistance mechanism of cisplatin against EC cells is important to provide new targets and strategies for treatment and prognosis of EC.

MicroRNAs (miRNAs) are a class of short and conserved non-coding RNAs that play a negative regulatory role at the post-transcriptional level by degrading target mRNAs or inhibiting RNA translation (3). A large number of studies have shown that many genes regulated by miRNAs are related to the response of tumor cells to chemotherapeutic drugs. The level of miR-21 was significantly increased in both tissue and serum samples from EC patients (4). It has been reported that miR-21 mediates the resistance of cholangio-carcinoma cells to HSP90 inhibitors by reducing the level of target gene DNAJB5 (5). In addition, miR-21 affected the sensitivity of esophageal cancer to cisplatin through targeting programmed cell death 4 (PDCD4) gene (6). Therefore, all these studies suggest that miR-21 is associated with drug resistance in tumor cells.

Recently, exosomes have been used as carriers for intercellular communication (7), as they can encapsulate substances such as miRNA and mRNA and exert biological effects in recipient cells (8). Expression of miR-21 was significantly up-regulated in exosomes of colon cancer cells compared with normal human colon epithelial cells. Moreover, miR-21 carried by exosomes of colon cancer could mediate the resistance to 5-fluorouracil by down-regulating PDCD4 (9). Thus, miR-21 may be a promising biomarker for diagnosis or drug resistance, but further *in vivo* and clinical studies are needed to verify it.

Fibroblasts are the key components of the tumor micro-environment (TME), have dynamic interactions with malignant cells, and are associated with cancer cells at all stages of cancer progression (10). A large number of studies have focused on the effects of drug resistance of cancer associated fibroblasts (CAFs), but few have concentrated on fibroblasts within TME. The specific mechanism of interaction between fibroblasts and ESCC needs further study.

In this study, the effect of human esophageal fibroblasts (HEF)-derived exosomal miR-21 on the cisplatin sensitivity of human esophageal squamous cells EC9706 was explored in order to further elucidate the mechanism of the drug-resistance mechanism of interaction between fibroblasts and ESCC.

## Material and Methods

### Cell lines and culture

EC9706 cells and HEF were purchased from the Institute of Cell Research, Chinese Academy of Sciences (China). These cells were cultured in RPMI 1640 medium (Gibco, USA) with penicillin-streptomycin (Solarbio, China) and 10% exosome-depleted fetal bovine serum (FBS; Gibco) in incubators containing 5% CO_2_ (Thermo Fisher Scientific, USA) at 37°C. HEF were cultured in the upper chamber and EC9706 cells in the lower chamber of a 24-well transwell system (Corning, USA) with a membrane pore size of 0.4 μm, and the HEF:EC9706 ratio was 3:1.

### Cell transfection

The antisense oligonucleotide sequence of miR-21-5p mimics (anti-miR-21-5p mimics) is 5′-AACAUCAGUCUGAUAAGCUAUU-3′. The antisense oligonucleotide sequence of miR-21-5p inhibitor (anti-miR-21-5p inhibitor) is 5′-UCAACAUCAGUCUGAUAAGCUA-3′, and the scrambled oligonucleotide sequence (anti-miR-NC) is 5′-ACGUGACACGUUCGGAGAATT-3′. The oligonucleotides were purified using high-performance liquid chromatography. Transfection of miR-21-5p mimics, miR-21-5p inhibitor, and miR-NC (negative control) (GenePharma, China) were performed using RFect siRNA/miRNA transfection reagent (Bio-Generation, China).

### Detection of cisplatin sensitivity against EC9706 cells

The cisplatin sensitivity against EC9706 cells was determined by the methyl thiazolyl tetrazollium (MTT) assay. Briefly, cells were seeded onto 96-well plates, and cultured for 48 h with different concentrations of cisplatin, and then incubated for 4 h with MTT (Solarbio). After that, formazan was dissolved with dimethyl sulfoxide (DMSO). The absorbance values were detected at 490 nm using a microplate reader (Biotek, USA) to calculate the half inhibition rate (IC_50_).

### Western blot and qRT-PCR analysis

Protein levels and changes were detected using western blot assays. Briefly, cells or exosomes were harvested at the specified time, and then prepared with SDS lysis buffer (Solarbio). Proteins (20 μg) were separated using a 10% polyacrylamide gel (Solarbio) and transferred to a 0.22-μm PVDF membrane (Merck Millipore, USA). The blots were blocked with 5% BSA (Boster, China) for 1 h at room temperature and incubated with primary antibody at 4°C overnight. Subsequently, horseradish peroxidase (HRP)-labeled secondary antibody was added, and the signals were observed using the ChemiDoc MP gel imaging system (Bio-Rad, USA). Reverse transcription was performed with 1 μg of total RNA using a HiScript II 1st strand cDNA synthesis kit (Vazyme, China). qRT-PCR was performed on a step-one plus real-time PCR system (Applied Biosystems, USA) using ChamQ universal SYBR qPCR master mix (Vazyme). All primers were synthesized by Suzhou Genewiz Biotech Co., Ltd. (China). Primer sequences were: miR-21, RT primer: 5′-TCGTATCCAGTGCAGGGTCCGAGGTGCACTGGATACGACTCAACATC-3′, forward primer: 5′-TGCGGTAGCTATCAGACTGATG-3′, reverse primer: 5′-CAGTGCAGGGTCCGAGGTAT-3′; U6, RT primer: 5′-CGCTTCACGAATTTGCGTGTCAT-3′, forward primer: 5′-GCTTCGGCAGCACATATACTAAAAT-3′, reverse primer: 5′-CCCTTCACGAATTTCCGTGTCAT-3′; PTEN, forward primer: 5′-TGGATTCGACTTAGACTTGACCT-3′, reverse primer: 5′-GGTGGGTTATGGTCTTCAAAAGG-3′; PDCD4, forward primer: 5′-GCAAAAAGGCGACTAAGGAAAAA-3-, reverse primer: 5′-TAAGGGCGTCACTCCCACT-3′; GAPDH, forward primer: 5′-GGAGCGAGATCCCTCCAAAAT-3′, reverse primer: 5′-GGCTGTTGTCATACTTCTCATGG-3′.

### Isolation and identification of exosomes

HEF were cultured in exosome-free RPMI1640 medium. When the cells were at 90% confluence, conditioned medium (CM) was collected and centrifuged at room temperature, 3000 *g* for 15 min. The collected supernatant was centrifuged at room temperature, 2500 *g* for 15 min in a 100-kD ultrafiltration centrifuge tube (Millipore, USA). A total of 500 µL of supernatant derived from the above step was centrifuged at 4°C, 17,500 *g* for 30 min, and then collected and centrifuged for 70 min at 4°C, 110,000 *g*. Consequently, the supernatant was discarded and phosphate-buffered saline (PBS) was added to resuspend the precipitation and was centrifuged for 70 min at 4°C, 110,000 *g* again. Finally, 100 μL PBS was added to resuspend the precipitate followed by filtering with 0.22-μm filters (EMD Millipore). The concentration of exosomes was detected using a bicinchoninic acid (BCA) protein concentration determination kit (Solarbio). The samples were dropped onto the copper mesh and negatively stained with 2% phosphotungstic acid for 2 min. After natural air drying for 15 min, the samples were observed under transmission electron microscopy (TEM; Jeol, Japan). Exosome particle size was detected using a dynamic light scattering system (DLS) (Malvin, UK). Exosome-specific proteins were detected by western blot.

### Exosomes uptake and transfer

The exosomes were resuspended with 250 μL of diluent C, and then 1 μL of PKH26 was added for 5 min at room temperature. Next, 250 μL fetal bovine serum was added to absorb the excess dye. The supernatant was discarded after centrifugation at 4°C, 110,000 *g* for 90 min. Subsequently, the supernatant was removed and then centrifuged at 4°C, 110,000 *g* for 90 min again. The precipitation was resuspended in 100 μL complete medium, and then sterilized by filtration through 0.22 μm filter membranes. EC9706 cells were direct co-cultured with the stained exosomes in a humidified atmosphere incubator containing 5% CO_2_ at 37°C for 24 h, and fixed with 4% formaldehyde solution for 10 min at room temperature. The cytoskeleton and nucleus were stained with TRITC phalloidin (Solarbio) and 4′,6-diamidino-2-phenylindole (DAPI; Solarbio), and then washed with PBS for three times. The uptake of HEF-derived exosomes by EC9706 cells were observed using a Cytation 5 microplate imager (Biotek, USA). To identify the transfer of exosomal miR-21-5p, FAM-tagged miR-21-5p was transfected into HEF. FAM-miR-21-5p-transfected HEF were co-cultured with EC9706 cells in 24-well transwell plates for 48 h. The cytoskeleton of EC9706 cells were stained with FITC phalloidin (Solarbio). Finally, the internalization of exosomal miR-21-5p was observed by the Cytation 5 microplate imager.

### Statistical analysis

All experimental data were analyzed using SPSS 22.0 (IBM, USA). The difference between two groups was determined by *t*-test, and the difference between three or more groups was analyzed by one-way ANOVA. P<0.05 indicated that the difference was statistically significant. All data are reported as means±SD. All experiments were repeated at least three times.

## Results

### Effects of miR-21-5p on the cisplatin sensitivity of EC9706 Cells

**Figure 1:**
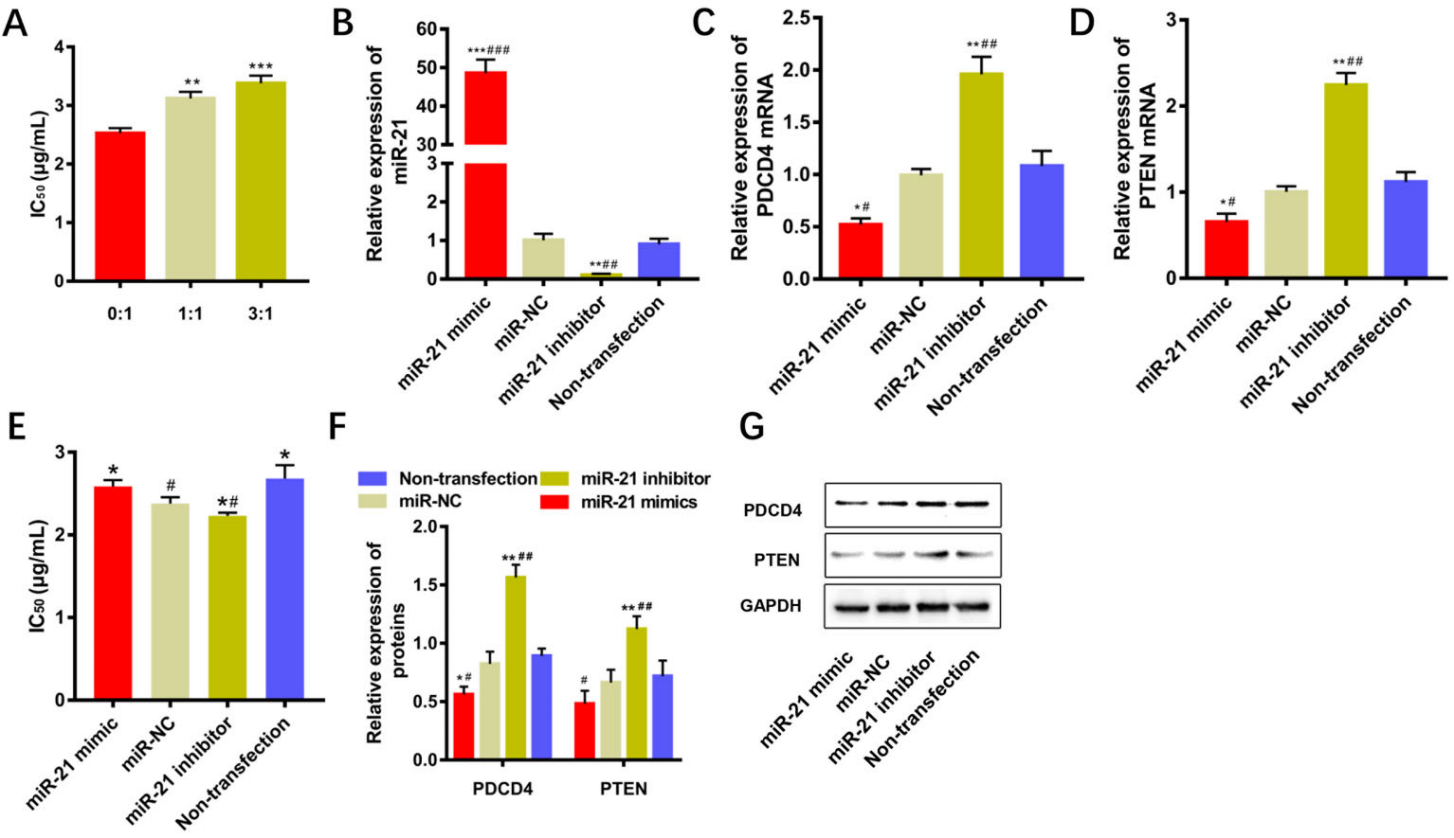
Effects of miR-21-5p on the sensitivity of EC9706 cells to cisplatin. **A**, The co-culture effect of human esophageal fibroblasts (HEF) with EC9706 on the sensitivity of EC9706 cells to cisplatin. **B**, **C**, and **D**, Expression level of miR-21-5p, PDCD4 mRNA, and PTEN mRNA in EC9706 cells. **E**, Changes of cisplatin sensitivity of EC9706 cells with different expression levels of miR-21-5p. **F** and **G**, Expression of related proteins at different expression levels of miR-21-5p in EC9706 cells. Data are reported as means±SD (n=3). *P<0.05, **P<0.01, ***P<0.001 compared with the miR-NC (negative control) group; ^#^P<0.05, ^##^P<0.01, ^###^P<0.001, compared with the non-transfection group (ANOVA).

After HEF was co-cultured with EC9706 for 48 h, the sensitivity of EC9706 cells to cisplatin was detected by MTT assay. The cisplatin IC_50_ values of the “3:1 co-culture group” (HEF:EC9706=3:1) increased compared with EC9706 alone (HEF:EC9706=0:1, P<0.001), suggesting that HEF co-cultured with EC9706 cells reduced the cisplatin sensitivity to EC9706 cells (Figure 1A). Compared with that of the miR-NC group, the miR-21-5p, PDCD4 mRNA, and PTEN mRNA in untransfected EC9706 cells showed no significant differences (P>0.05, Figure 1B-D). Compared with that of the miR-NC group or non-transfection group, the content of miR-21-5p was significantly increased in EC9706 cells transfected with miR-21-5p mimics (P<0.001), and significantly decreased (P<0.05) in EC9706 cells transfected with miR-inhibitor (Figures 1B). Furthermore, the content of PDCD4 mRNA and PTEN mRNA were significantly decreased in EC9706 cells transfected with miR-21-5p mimics (P<0.05), and significantly increased in EC9706 cells transfected with miR-inhibitor (Figure 1C and D). Compared with the miR-NC group, cisplatin IC_50_ value was increased in the miR-21-5p overexpression group (P<0.05) and decreased in the miR-21-5p inhibitor group (P<0.05, Figure 1E). In addition, the overexpression of miR-21-5p decreased the expression of PDCD4 protein (P<0.05) and PTEN protein (P<0.05) in EC9706 cells (Figure 1F and G), suggesting that the overexpression of miR-21-5p can inhibit the expression of PDCD4 and PTEN protein in EC9706 cells and can decrease the cisplatin sensitivity to EC9706 cells.

### HEF-derived exosomal miR-21-5p affected the EC9706 cells

**Figure 2:**
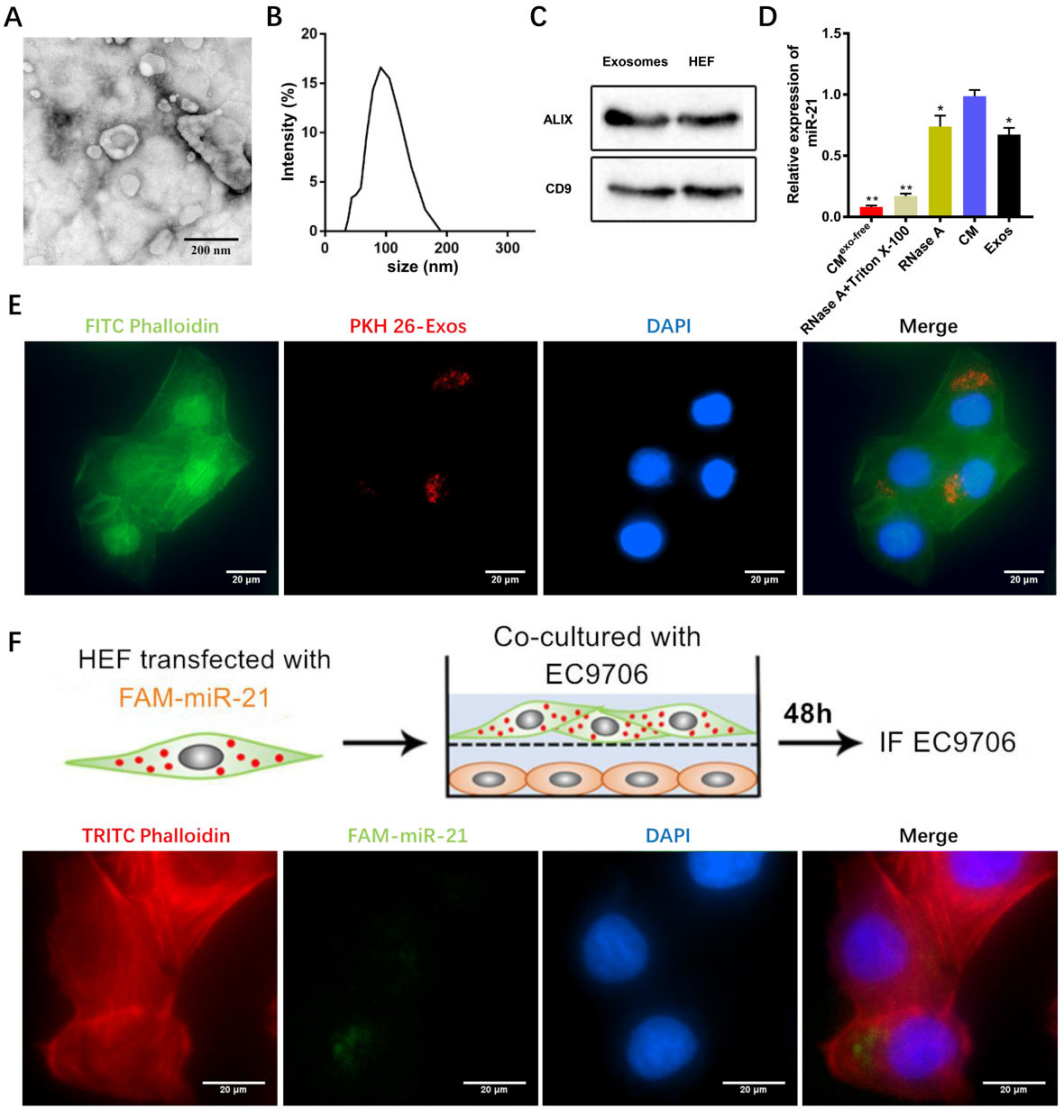
A, The morphology of human esophageal fibroblasts (HEF)-derived exosomes under transmission electron microscopy (scale bar, 200 μm). **B**, The size and intensity of extracted exosomes. **C**, The expression of the hallmark proteins ALIX and CD9 with western blot detection. **D**, The miR-21-5p expression levels of each component in conditioned medium (CM). **E**, Detection of PKH26-labeled HEF-derived exosomes (Exos) by Cytation 5 microplate imager (scale bar, 20 μm, red signal). **F**, HEF were transfected with FAM-miR-21-5p transiently, and then co-cultured with EC9706 cells for 48 h. The green FAM-miR-21-5p signal was detected by Cytation 5 microplate imager (scale bar, 20 μm). Data are reported as means±SD (n=3), *P<0.05, **P<0.01, compared with the CM group (ANOVA).

After collection with ultracentrifugation, exosome morphology was observed under TEM to be a saucer-shaped concave vesicle (magnification: 50,000×, Figure 2A). The particle size of vesicles was measured by DLS, and measured between 30 to 180 nm (Figure 2B). The results of HEF-derived exosomes specifically expressed TSPAN29 (CD9) and Programmed Cell Death 6 Interacting Protein (ALIX) proteins by western blot (Figure 2C), further confirming that the vesicle-like components extracted from CM were HEF exosomes. qRT-PCR showed that compared with the CM group, the level of miR-21-5p in CM treated with both membrane breakers Triton X-100 and RNase A significantly decreased. It suggested that the miR-21-5p in CM may be encapsulated by exosomes, which are coated with a membrane-structured substance, and then protected by miR-21-5p from RNase A degradation (Figure 2D). After C9706 cells were co-incubated with PKH26 dyed exosomes for 24 h, the results showed that HEF-derived exosomes were phagocytosed and taken-up by EC9706 cells (Figure 2E). As shown in the pattern diagram, FAM-tagged miR-21-5p (FAM-miR-21-5p) was transfected into HEF, and then taken-up into co-cultured EC9706 cells in the lower chamber of the transwell system after 48 h. The observation with microplate imaging showed that green fluorescent signals appeared in co-cultured EC9706 cells suggesting that FAM-tagged miR-21-5p in HEF was captured by EC9706 cells through exosomes (Figure 2F).

### HEF-derived exosomal miR-21-5p reduced the cisplatin sensitivity to EC9706 cells

**Figure 3:**
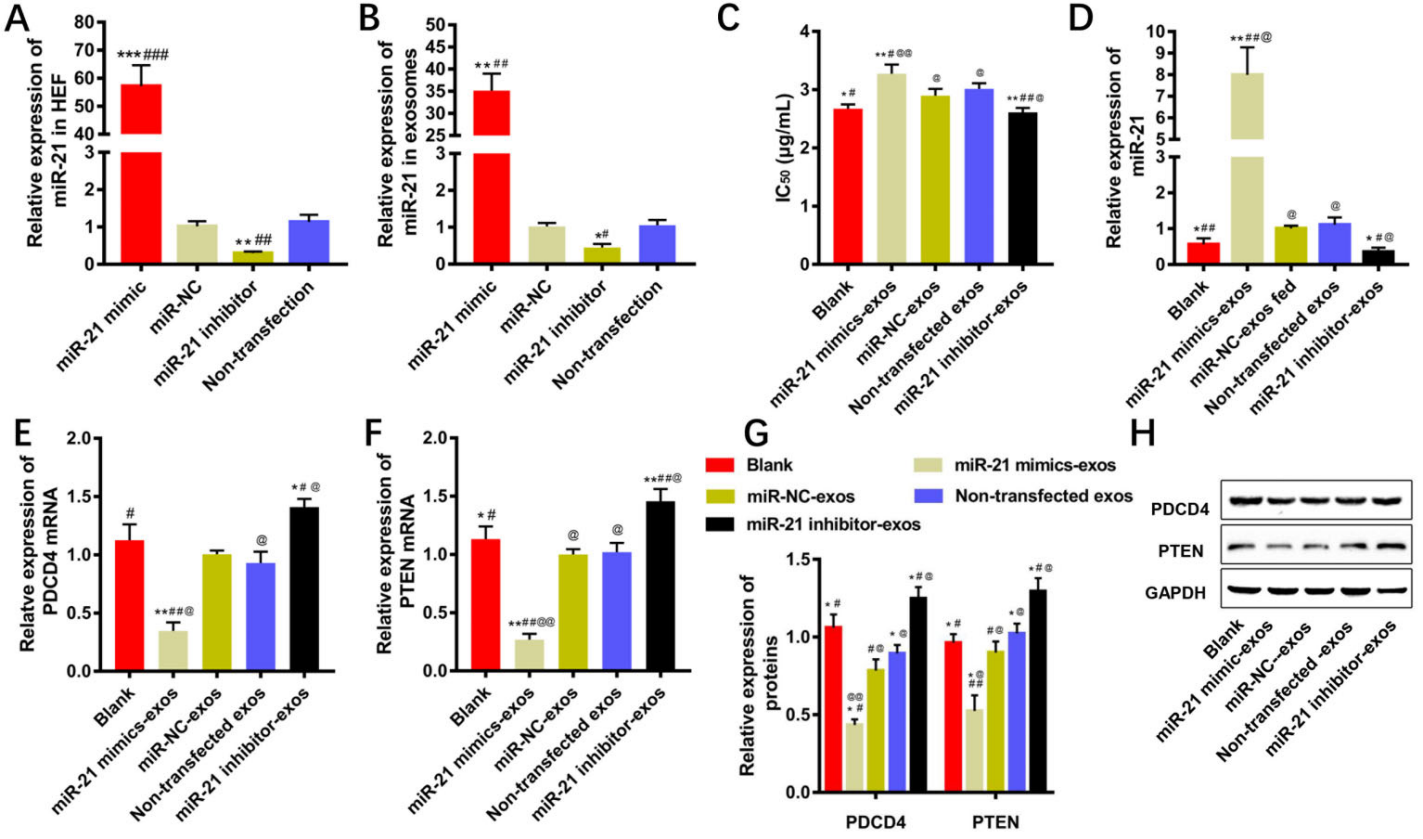
Effects of the human esophageal fibroblast (HEF)-derived exosomal miR-21-5p on cisplatin sensitivity in EC9706 cells. **A**, The miR-21-5p expression levels in HEF with qRT-PCR analysis. **B**, The miR-21-5p expression levels of each component in HEF-derived exosomes with qRT-PCR analysis. **C**, The change of cisplatin sensitivity of EC9706 cells after being fed with HEF-derived exosomes (exos). **D**-**F**, qRT-PCR results of miR-21-5p, PDCD4 mRNA, and PTEN mRNA expression in EC9706 cells. **G** and **H**, The expression level of related proteins in EC9706 cells by western blot detection. Data are reported as means±SD (n=3), *P<0.05, **P<0.01, ***P<0.001 compared with the miR-NC (negative control) group; ^#^P<0.05, ^##^P<0.01, ^###^P<0.001 compared with the non-transfection group; ^@^P<0.05, ^@@^P<0.01 compared with the blank group (ANOVA).

Each CM derived from HEF culture was collected and HEF-derived exosomes were extracted. After transfection, the content of miR-21-5p both in the miR-21-5p mimics-transfected HEF and HEF-derived exosomes were significantly increased. In contrast, the content of miR-21-5p was significantly decreased both in miR-21-5p inhibitor-transfected HEF and its exosomes compared with that of miR-NC-transfected HEF and its exosomes, respectively (P<0.01, Figure 3A and B). EC9706 cells were co-cultured with HEF-derived exosomes with different treatments, and the change of cisplatin sensitivity to EC9706 cells was detected by MTT assay. Compared with the miR-NC-exos-transfected group (i.e., HEF-derived exosomes transfected with miR-NC into EC9706 cells), the IC_50_ value of the miR-21-5p mimics-exos-transfected group was significantly increased (P<0.01, Figure 3C). Results of qRT-PCR demonstrated that compared with the corresponding indicators of the miR-NC-exos-transfected group, the content of miR-21-5p was significantly increased (P<0.01), and the content of either PDCD4 mRNA or PTEN mRNA was decreased (P<0.01) in the miR-21-5p mimics-exos-transfected group (Figure 3D-F). Compared with the corresponding indicators in the miR-NC-exos-transfected group, both the content of PDCD4 and PTEN proteins were decreased (P<0.05) in the miR-21-5p mimics-exos-transfected group (Figure 3G and H).

## Discussion

Owing to the early-stage symptoms of EC being often not evident, most patients lose the best chance of surgical resection. Middle and advanced stages of EC are generally treated by chemotherapy and radiation. Cisplatin is a common chemotherapeutic drug used to treat esophageal cancer. However, cisplatin resistance of esophageal cancer cells affects the chemotherapeutic effects resulting in a poor prognosis in patients with either ESCC or esophageal adenocarcinoma, and the 5-year survival rate of patients is less than 20% (11). miR-21 plays an important role in promoting tumor proliferation, angiogenesis, epithelial-mesenchymal transition, and chemoresistance. PTEN is the first discovered tumor suppressor gene with bispecific phosphatase activity and inhibition of tumor growth (12). PTEN, as a direct target gene of miR-21, was significantly downregulated in gemcitabine-resistant breast cancer cells (13). It can be seen that PTEN and PDCD4, as tumor suppressors, can not only inhibit the growth and invasion of tumors, but also downregulate the 5-fluorouracil resistance induced by miR-21 in pancreatic cancer cells (14). Similarly, our results showed that miR-21 works through the target genes PTEN and PDCD4, which is consistent with the results of rectal cancer research (15).

Although various mechanisms of drug resistance have been reported, such as drug efflux, increased DNA repair, and insensitivity to drug-induced apoptosis, etc, recent studies have shown that drug resistance of tumor cells is mainly caused by changes in its TME rather than in itself (16). At present, most studies focus on the tumor-related stromal cells in TME. These cells are mostly primary culture cells obtained from isolation of clinical pericancerous tissues. Because these cells live together with tumor tissues for a long time, they exhibit some malignant phenotypes. Compared with fibrocytes, head and neck tumor-derived CAFs showed significantly enhanced tolerance to cisplatin (17). However, it was found that under indirect co-culture mode, adipocytes could induce epithelial-mesenchymal transition of thyroid cancer cells and promote drug tolerance of thyroid cancer cells to docetaxel and cisplatin (18). Similarly, this study found that indirect co-culture of HEF with EC9706 cells resulted in decreased cytotoxicity of cisplatin to EC9706 cells and increased expression of miR-21-5p in EC9706 cells. Indeed, tumor growth is not just determined by malignant cancer cells themselves, but also by the tumor stroma (19). These results implied that the microenvironment may be remodeled in the context of interactions between tumor cells and surrounding normal stromal cells, which in turn facilitates chemoresistance. The direction of change in this context is a complex process, and different stages of tumorigenesis or different responses to different treatments will be reported elsewhere.

Recent studies have shown that exosomes promote tumor progression and increase drug resistance of cancer cells by delivering oncogenic DNA, proteins, and non-coding RNAs (20). Our study found that miR-21-5p with oncogene properties in CM was mainly present in exosomal vesicles, and the main pathway of promoting cisplatin tolerance in EC9706 cells by miR-21-5p resulted from the mediation by HEF-derived exosomes. It may transmit malignant signals from stromal cells to tumor cells, thus promoting the chemoresistance of tumor cells.

In conclusion, after EC9706 cells were co-cultured with HEF-derived exosomal miR-21, the expressions of PTEN and PDCD4 were inhibited significantly in EC9706 cells, which ultimately led to a decreased cisplatin sensitivity of EC9706 cells. Inhibition of fibroblast-derived exosomal miR-21 may provide new treatment targets and strategies for ESCC treatments.

## References

[1] Smyth EC, Lagergren J, Fitzgerald RC, Lordick F, Shah MA, Lagergren P (2017). Oesophageal cancer. Nat Rev Dis Primers.

[2] Hong L, Han Y, Lu Q, Zhang H, Zhao Q, Wu K (2012). Drug resistance-related microRNAs in esophageal cancer. Expert Opin Biol Ther.

[3] Dong H, Lei J, Ding L, Wen Y, Ju H, Zhang X (2013). MicroRNA: function, detection, and bioanalysis. Chem Rev.

[4] Lv H, He Z, Wang H, Du T, Pang Z (2016). Differential expression of miR-21 and miR-75 in esophageal carcinoma patients and its clinical implication. Am J Transl Res.

[5] Lampis A, Carotenuto P, Vlachogiannis G, Cascione L, Hedayat S, Burke R (2018). MIR21 drives resistance to heat shock protein 90 inhibition in cholangiocarcinoma. Gastroenterology.

[6] Yang YC, Liu GJ, Yuan DF, Li CQ, Xue M, Chen LJ (2019). Influence of exosome-derived miR-21 on chemotherapy resistance of esophageal cancer. Eur Rev Med Pharmaco Sci.

[7] Valadi H, Ekstrom K, Bossios A, Sjostrand M, Lee JJ, Lotvall JO (2007). Exosome-mediated transfer of mRNAs and microRNAs is a novel mechanism of genetic exchange between cells. Nat Cell Biol.

[8] Melo SA, Sugimoto H, O'Connell JT, Kato N, Villanueva A, Vidal A (2014). Cancer exosomes perform cell-independent microRNA biogenesis and promote tumorigenesis. Cancer Cell.

[9] Sun LH, Tian D, Yang ZC, Li JL (2020). Exosomal miR-21 promotes proliferation, invasion and therapy resistance of colon adenocarcinoma cells through its target PDCD4. Sci Rep.

[10] Kalluri R, Zeisberg M (2006). Fibroblasts in cancer. Nat Rev Cancer.

[11] Chen W, Zheng R, Baade PD, Zhang S, Zeng H, Bray F (2016). Cancer statistics in China, 2015. CA Cancer J Clin.

[12] Smith U (2012). PTEN--linking metabolism, cell growth, and cancer. N Engl J Med.

[13] Wu ZH, Tao ZH, Zhang J, Li T, Ni C, Xie J (2016). MiRNA-21 induces epithelial to mesenchymal transition and gemcitabine resistance via the PTEN/AKT pathway in breast cancer. Tumour Biol.

[14] Wei X, Wang W, Wang L, Zhang Y, Zhang X, Chen M (2016). MicroRNA-21 induces 5-fluorouracil resistance in human pancreatic cancer cells by regulating PTEN and PDCD4. Cancer Med.

[15] Amirhossein B, Melika R, Afsane B, Majid K, Hamid F, Mikhail R (2019). Diagnostic, prognostic, and therapeutic potency of microRNA 21 in the pathogenesis of colon cancer, current status and prospective. J Cell Physiol.

[16] Santos P, Almeida F (2020). Role of exosomal miRNAs and the tumor microenvironment in drug resistance. Cells.

[17] Qin X, Guo H, Wang X, Zhu X, Yan M, Wang X (2019). Exosomal miR-196a derived from cancer-associated fibroblasts confers cisplatin resistance in head and neck cancer through targeting CDKN1B and ING5. Genome Biol.

[18] Fei S, Ahn S, Saha A, DiGiovanni J, Koloni MG (2019). Adipose stromal cell targeting suppresses prostate cancer epithelial-mesenchymal transition and chemoresistance. Oncogene.

[19] Kalluri R (2003). Basement membranes: structure, assembly and role in tumour angiogenesis. Nat Rev Cancer.

[20] Janas T, Janas MM, Sapon K, Janas T (2015). Mechanisms of RNA loading into exosomes. FEBS Lett.

